# Patterns of Congenital Malformations in a Tertiary Newborn Care Unit in Central India: Implications for Prenatal Care

**DOI:** 10.7759/cureus.86561

**Published:** 2025-06-22

**Authors:** Ravi Ambey, Richa Ambey, Kamal S Bhadauria

**Affiliations:** 1 Pediatrics, Gajra Raja Medical College, Gwalior, IND; 2 Dermatology, Gajra Raja Medical College, Gwalior, IND

**Keywords:** birth defects, congenital abnormalities, congenital anomalies, congenital malformations, live births, pattern, prenatal care, sick newborn care unit, still birth

## Abstract

Background: Congenital malformations pose emotional strains for expecting parents and are linked to stillbirths, newborn deaths, and post-neonatal deaths. This understanding is crucial for prenatal counseling and risk assessment in maternal and child healthcare. This study provides an overview of congenital malformation patterns in a tertiary care neonatal unit in central India. Understanding the burden and pattern of congenital malformation is key in monitoring the trend and prevention of stillbirths, especially those in low‐income countries.

Methods: This was a prospective, observational study that recruited all neonatal unit admissions with major congenital malformations, with the exceptions of newborns without known biological mothers and those whose parents refused consent, during a one-year span from May 2024 to April 2025. Parental demographics, newborn traits, and outcome measures such as prior stillbirth, birth weight, gestational age, and viral infections were analysed.

Results: Among 4407 admissions, 200 newborns had congenital malformations (4.5%), which included gastrointestinal (45%; n=90), CNS (25%; n=50), cardiovascular (10%; n=20), and musculoskeletal (7%; n=14) malformations. Of the cohort, 56% were male and 44% female; 154 (76%) were full term, 44 (22%) were preterm, and 4 (2%) were post-term births, with 130 (65%) being low birth weight. A total of 70 infants died in the neonatal period, with a case fatality ratio of 32% (n=16) from CNS, 25% (n=5) from cardiovascular, and 18.88% (n=17) from gastrointestinal malformations. Previous history of abortion and stillbirth was associated with 30% (n=60) and 9% (n=18) of congenital malformation, respectively.

Conclusion: The study highlights the burden of congenital malformation in a tertiary care sick newborn care unit in central India. Antenatal care, including screening for congenital malformation, counselling, and management, will help to decrease the burden of congenital malformation. Further research is essential to refine interventions and support families affected by these challenges.

## Introduction

Congenital malformations and stillbirth are two distressing realities in obstetrics, each carrying its own set of challenges and emotional burdens for expecting parents. According to the WHO, the definition of stillbirth for international comparison is a baby born with no signs of life at 28 completed weeks of gestation or later, or weighing 1,000 grams or more [1]. The relationship between congenital malformation and previous history of stillbirths can be complex and multifaceted, and the understanding of this link has important implications for prenatal counselling, risk assessment, and preventive strategies in maternal and child healthcare.

As per the WHO, congenital malformation is defined as structural or functional abnormalities that occur during intrauterine life and can be identified prenatally, at birth, or sometimes may only be detected later in infancy [2]. Globally, an estimated 7.9 million infants are born with congenital malformations and are at risk of physical impairment after birth, and perinatal mortality. Ninety-four percent of these defects are seen in low-resource regions. Certain factors contribute to this higher risk, like a lack of sufficient nutritious food for pregnant women, exposure to agents or factors such as infection and alcohol, and poor access to health care and screening. An estimated 240,000 newborns die worldwide within 28 days of birth annually due to congenital malformations. In addition, congenital malformations cause the death of 170,000 children between the ages of one month and five years [2].

Congenital malformations are caused by various factors, including single-gene disorders, chromosomal abnormalities, multifactorial transmission, teratogen exposure, and micronutrient deficiencies. Maternal diabetes, infections such as cytomegalovirus, rubella, alcohol or tobacco consumption, environmental contaminants like pesticides, and radiation are also important risk factors. Congenital malformation affects individuals, families, society, and the healthcare system severely and in the long term [3].

There is a need for improved understanding of the epidemiology of congenital malformations, which will help align focused efforts to prevent them. This study provides an overview of the congenital malformation patterns in a tertiary care referral sick newborn care unit in central India, and studies the association with potential risk factors, such as previous adverse fatal outcomes, including stillbirths. The study will help stakeholders to focus on India-specific data to tackle the problem.

## Materials and methods

This was a facility-based, prospective, observational, descriptive study conducted in the Sick Newborn Care Unit of Kamlaraja Hospital, Gajra Raja Medical College, Gwalior, Madhya Pradesh, India, from May 2024 to April 2025. The study was approved by the Institutional Ethical Committee, Gajra Raja Medical College (approval number: 1375/IEC-GRMC/2024, dated May 1, 2024).

Eligibility criteria

All consecutive hospitalized newborns with major congenital malformations were included in the study. Major malformations are defined as malformations that are life-threatening, require surgery, or present a significant disability. Newborns without known biological mothers and those whose parents refused consent were excluded from the study.

Data collection

Data were collected from families after obtaining informed written consent, and strict confidentiality was maintained while processing the data and writing the observation by eliminating all personal identifiers. Various malformations were recorded based on physical examination, relevant investigations, and imaging. They were classified on the basis of clinical judgement and appropriate imaging methods. Birth weight, comorbid conditions, as well as outcomes, were recorded. Maternal information such as maternal parity, gestational age, education, socioeconomic status, previous history of congenital malformation, previous history of stillbirths and abortions, concomitant medical conditions, and TORCH (toxoplasmosis, rubella, cytomegalovirus, herpes simplex, and HIV) infections in the first trimester were also recorded. Antenatal records, where available, were also considered for extracting information.

Statistical analysis

The frequency and pattern of malformations, male-to-female ratio, relation with birth weight and gestational age, and final outcome were noted as outcome variables. Statistical analysis was done with IBM SPSS Statistics for Windows, version 29 (Released 2022; IBM Corp., Armonk, New York, United States).

## Results

Among 4407 newborn admissions during the study period, 200 newborns had congenital malformations, which constituted 4.5% of the total admissions. The study cohort consisted of 56% male (n=112) and 44% female (n=88) infants; 76% (n=152) were full term, 21% (n=44) were preterm, and 2% (n=4) were post-term, with 65% (n=130) being low birth weight. Of the infants with congenital malformations in the study, 55% (n=110) were from rural areas and 60% (n=120) were born by normal vaginal delivery (Table 1).

**Table 1:** Baseline characteristics of study subjects (N=200)

Characteristic	Variables	Frequency	Percentage
Sex	Male	112	56%
	Female	88	44%
Gestation	Full term	154	76%
	Preterm	44	22%
	Post term	4	2%
Birth weight	Normal birth weight	70	35%
	Low birth weight	130	65%
Residence	Rural	110	55%
	Urban	90	45%
Mode of delivery	Normal vaginal delivery	120	60%
	Cesarean section	80	40%

Table 2 depicts the distribution of patterns of congenital malformations; gastrointestinal malformations were 45% (n=90), central nervous system (CNS) malformations were 25% (n=50), cardiovascular malformations were 10% (n=20), musculoskeletal were 7% (n=14), and genitourinary malformation were 2% (n=4). The most common individual congenital malformation was tracheoesophageal fistula (n=56, 28%), followed by congenital hydrocephalus (n=26, 13%), neural tube defects (n=24, 12%), and cleft lip and palate (n=18, 9%). Mortality rate in infants with congenital malformations in the neonatal period was 35% (n=70), with a case fatality rate of 32% (n=16) from CNS, 25% (n=5) from cardiovascular, and 18.88% (n=17) from gastrointestinal malformations.

**Table 2:** Distribution of pattern of congenital malformation (N=200)

Malformations		Frequency	Percentage
Gastrointestinal malformation		90	45%
1.	Tracheoesophageal fistula	56	
2.	Anorectal malformation	14	
3.	Gastroschisis	7	
4.	Omphalocele	4	
5.	Umbilical hernia	3	
6.	Duodenal atresia	2	
7.	Hirschsprung disease	2	
8.	Ileal atresia	2	
Central nervous system malformation		50	25%
1.	Congenital hydrocephalus	26	
2.	Neural tube defect	24	
Cardiovascular malformation		20	10%
1.	Ventricular septal defect	5	
2.	Atrial septal defect	4	
3.	Complex congenital heart disease	4	
4.	Patent ductus arteriosus	4	
5.	Transposition of great arteries	3	
Musculoskeletal malformation		14	7%
1.	Congenital talipes equinovarus	8	
2.	Congenital diaphragmatic hernia	4	
3.	Congenital dislocation of hip	2	
Genitourinary malformation		4	2%
1.	Pelvo-ureteric junction (PUJ) obstruction	4	
Miscellaneous		22	11%
1.	Cleft lip and palate	18	
2.	Pierre Robin syndrome	2	
3.	Down syndrome	2	

**Figure 1:**
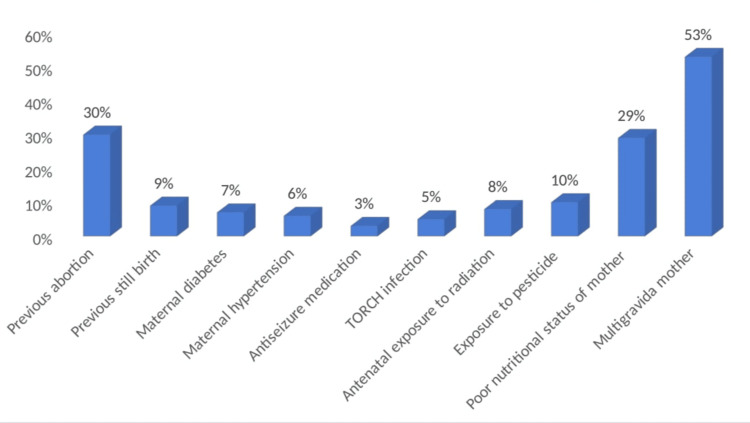
Maternal risk factors in study population TORCH: toxoplasmosis, rubella, cytomegalovirus, herpes simplex, and HIV

Figure 1 depicts the maternal risk factors in the study population. It is noteworthy to mention that the majority of congenital malformations were present among neonates born to multigravida mothers (53%, n=106). Some other risk factors noted were poor nutritional status of mother, i.e. BMI <18.5 kg/m^2^ (29%, n=58), maternal diabetes (7%, n=14), maternal hypertension (6%, n=12), history of antiseizure medication (3%, n=6), TORCH infection (5%, n=10), exposure to radiation (8%, n=16), and exposure to pesticides (10%, n=20). Strikingly, a previous history of abortion and stillbirths was observed in 30% (n=60) and 9% (n=18) of mothers, respectively.

Table 3 shows the association of congenital malformation with baseline variables. The chi-square test was used. No significant association of congenital malformation with baseline variables (p>0.05) was observed.

**Table 3:** Association of congenital malformations with baseline variables χ2, chi-square value p value <0.05 significant GI: gastrointestinal; CNS: central nervous system

Variables		GI malformation, n (%)	CNS malformation, n (%)	Cardiovascular malformation, n (%)	Musculoskeletal malformation, n (%)	Genitourinary malformation, n (%)	Miscellaneous, n (%)	χ^2^	P value
Sex	Male	46 (41.1%)	29 (25.9%)	14 (12.5%)	8 (7.1%)	2 (1.8%)	13 (11.6%)	2.6	0.62
	Female	44 (50%)	21 (23.9%)	6 (6.8%)	6 (6.8%)	2 (2.3%)	9 (10.2%)		
Gestation	Full term	66 (43.4%)	39 (25/7%)	18 (11.8%)	11 (7.2%)	3 (2%)	15 (9.9%)	3.8	0.49
	Preterm	21 (47.7%)	10 (22.7%)	2 (4.5%)	3 (6.8%)	1 (2.3%)	7 (15.9%)		
	Post term	3 (75%)	1 (25%)	0 (0)	0 (0)	0 (0)	0 (0)		
Birth weight	Normal birth weight	26 (37.1%)	19 (27.1%)	9 (12.9%)	7 (10%)	1 (1.4%)	8 (11.4%)	3.3	0.51
	Low birth weight	64 (49.2%)	31 (23.8%)	11 (8.5%)	7 (5.4%)	3 (2.3%)	14 (10.8%)		
Residence	Rural	45 (40.9%)	31 (28.2%)	11 (10%)	9 (8.2%)	2 (1.8%)	12 (10.9%)	2.2	0.70
	Urban	45 (50%)	19 (21.1%)	9 (10%)	5 (5.6%)	2 (2.2%)	10 (11.1%)		
Mode of delivery	Normal vaginal delivery	51 (42.5%)	34 (28.3%)	11 (9.2%)	8 (6.7%)	3 (2.5%)	13 (10.8%)	1.9	0.74
	Caesarean section	39 (48.8%)	16 (20%)	9 (11.3%)	6 (7.5%)	1 (1.3%)	9 (11.3%)		

Table 4 depicts the association of various risk factors with congenital malformations. The chi-square test was used. A significant association of the multigravida status of mothers was observed with gastrointestinal malformations (p<0.05). There was no significant association of other risk factors with congenital malformation (p>0.05).

**Table 4:** Association of various risk factors with congenital malformations χ2, chi-square value p value <0.05 significant GI: gastrointestinal; CNS: central nervous system; TORCH: toxoplasmosis, rubella, cytomegalovirus, herpes simplex, and HIV

Risk factors	GI malformation, n (%)	CNS malformation, n (%)	Cardiovascular malformation, n (%)	Musculoskeletal malformation, n (%)	Genitourinary malformation, n (%)	Miscellaneous, n (%)	χ^2^	P value
Previous abortion	32 (53.3%)	12 (20%)	3 (5%)	3 (5%)	1 (1.7%)	9 (15%)	6.1	0.19
Previous still birth	9 (50%)	3 (16.7%)	2 (11.1%)	1 (5.6%)	1 (5.6%)	2 (11.1%)	0.8	0.94
Maternal diabetes	8 (57.1%)	2 (14.3%)	1 (7.1%)	1 (7.1%)	1 (7.1%)	1 (7.1%)	1.9	0.74
Maternal hypertension	8 (66.7%)	1 (8.3%)	1 (8.3%)	0 (0)	1 (8.3%)	1 (8.3%)	2.8	0.58
Antiseizure medication	2 (33.3%)	2 (33.3%)	1 (16.7%)	0 (0)	0 (0)	1 (16.7%)	0.9	0.92
TORCH infection	3 (30%)	3 (30%)	2 (20%)	1 (10%)	0 (0)	1 (10%)	1.7	0.78
Antenatal exposure to radiation	6 (37.5%)	4 (25%)	2 (12.5%)	2 (12.5%)	1 (6.3%)	1 (6.3%)	2.5	0.64
Exposure to pesticide	8 (40%)	8 (40%)	2 (10%)	2 (10%)	0 (0)	0 (0)	2.7	0.61
Poor nutritional status of mother	28 (48.3%)	13 (22.4%)	2 (3.4%)	2 (3.4%)	1 (1.7%)	12 (20.7%)	6.6	0.08
Multigravida mother	61 (57.5%)	22 (20.8%)	6 (5.7%)	4 (3.8%)	1 (0.9%)	12 (11.3%)	18.3	0.001

## Discussion

The present study observed a 4.5% prevalence of congenital malformations in hospitalized newborns, which is comparable with other Indian studies such as those by Sravani et al. in Andaman and Nicobar Islands (3.7%) [4], Tirumani and Khatija in Hyderabad (3%) [5], Seba et al. in Bhubaneswar, Odisha (2.9%) [6], Tiwari and Gupta in North India (2.56%) [7], and Ghosh et al. in Midnapore, West Bengal (2.14%) [8]. It is also comparable with reports from different parts of the world, such as the studies by Anane-Fenin et al. in Ghana (2.8%) [9] and Tomatir et al. in Turkey (2.9%) [10].

The incidence of congenital malformation was higher in male newborns compared to female newborns (56% vs 44%) with a sex ratio of 1.27, which is comparable with El Koumi et al.'s study, which depicted a gender ratio of 1.09 [11]. Of the infants with congenital malformation in the study, 65% were noted to be of low birth weight. Taksande et al. also observed low birth weight as a risk factor for the occurrence of congenital malformations [12]. However, low birth weight may be a cause or a consequence of congenital malformation. The majority of study subjects were born to multiparous mothers, which is in alignment with the findings reported by Seba et al. [6], Sarkar et al. [13], and Bhalerao and Bhalerao [14].

The present study noticed highest incidence of malformations was gastrointestinal in nature (45%), which is consistent with Tirumani et al.'s study, which reported the highest malformations in the gastrointestinal system (28.8%) [5]. On the other hand, Seba et al. [6], Cherian et al. [15], and Bhalerao and Bhalerao [14] observed the highest incidence of malformations in the musculoskeletal system, of 31.6%, 9.69 per 1000 live births, and 33.2%, respectively. Shrestha and Shrestha [16] noted the highest incidence in genitourinary malformation (24.2%), and Shravani et al. [4] noted the highest incidence in cardiovascular malformations (44.3%).

Many risk factors are correlated with the occurrence of congenital malformations. Previous history of abortions (30%) and previous history of stillbirths (9%) were observed in the present study. Singh and Sinha observed a significant association of previous abortion with the occurrence of congenital malformations [17]. In our cohort, poor nutritional status of the mother (BMI< 18.5 kg/m^2^), maternal diabetes, maternal hypertension, anti-seizure medications, TORCH infection during pregnancy, and exposure to radiation and pesticides were observed to be risk factors. Alam et al. found parental consanguinity, maternal undernutrition, obesity, history of abnormalities in the family, low birth weight, and preterm birth were associated with a higher incidence of congenital malformation [3]. One of the novel findings in the present study was that a significant association was found between the multigravida status of the mother and gastrointestinal malformation. This study also tried to observe the association of a previous history of abortion and stillbirth in mothers, which has not been studied in most of the studies, including one recent study from Nigeria by Omar et al. [18].

Certain limitations are seen in the present study. This study is a facility-based assessment of congenital malformation patterns in a tertiary newborn care unit, which might not truly reflect the actual congenital malformation pattern in the community. Some of the factors affecting the congenital malformation at the facility are intramural vs extramural delivery, severity of malformation, parents’ perception of malformation, factors related to the referral system, and institutional services for management of that particular malformation (i.e., neurosurgical, paediatric surgical services, and cardiovascular services, etc.). Further diagnostic limitations include the lack of genetic testing and autopsy confirmation, which were not possible in the present study. 

## Conclusions

Congenital malformations contribute to neonatal mortality as well as infant mortality and under-five mortality. Congenital malformations also exert long-lasting and irreversible negative impacts on individuals, families, society, and the healthcare system. Antenatal care, including screening, folic acid supplementation, counselling, and management, will help to decrease the burden of congenital malformation.

The study results show that congenital malformations were more common when there was a previous history of abortion and or stillbirths. Congenital malformations were more common in males and low birth weight babies. Gastrointestinal and CNS malformations were most frequent. The multigravida status of the mother was significantly associated with the occurrence of gastrointestinal malformations.
